# Osteoblastic Osteosarcoma Arising beneath an Osteochondroma in an 11-Year-Old Male with Multiple Hereditary Exostoses

**DOI:** 10.1155/2018/8280415

**Published:** 2018-07-12

**Authors:** Emmanuel Bukara, Alex M. Buteera, Robert Karakire, Felix Manirakiza, Samuel Muhumuza, Emmanuel Rudakemwa, Lynnette Kyokunda

**Affiliations:** ^1^Department of Orthopaedic Surgery, King Faisal Hospital, OSHEN, Kigali, Rwanda; ^2^Department of Pathology & Laboratory Medicine, King Faisal Hospital, OSHEN, Kigali, Rwanda; ^3^Department of Anaesthesia, King Faisal Hospital, OSHEN, Kigali, Rwanda; ^4^Department of Radiology, King Faisal Hospital, OSHEN, Kigali, Rwanda

## Abstract

**Introduction:**

Multiple hereditary exostoses (MHE) is a rare autosomal dominant disorder characterized by the presence of multiple skeletal deformities. They are painless slow-growing lesions. Malignant transformation tends to occur later in adulthood and has only been seen in 1–5% of patients.

**Objective:**

We describe the clinical, radiological, and pathological characteristics of a child with MHE who developed osteoblastic osteosarcoma beneath an osteochondroma.

**Case Presentation:**

An 11-year-old male Rwandan presented to our hospital with a two-week history of a dull persistent pain in his left distal femur and loss of weight and appetite. There was no relief with pain killers. He was a known case of multiple hereditary exostoses diagnosed at age 3. He began experiencing mild symptoms 6 months prior to admission which worsened in the last two weeks prior to his admission. On examination, he had multiple palpable bony swellings bilaterally on the proximal humeri and distal femurs. X-rays showed multiple exostoses and MRI showed a lesion with heterogeneous signal intensities that suggested malignant transformation. At surgery, a necrotic lesion beneath the exostosis was excised and sent for histopathological analysis which confirmed osteochondroma with an osteoblastic osteosarcoma in the marrow cavity. Chemotherapy and limb-salvaging surgery were done and he has recovered well.

**Conclusion:**

Osteosarcomas arising at sites of MHE have not been previously reported in Africa. These tumors rarely undergo malignant transformation.

## 1. Introduction

Multiple hereditary exostoses (MHE) also referred to as diaphyseal aclasis or familial osteochondromatosis is a rare autosomal dominant skeletal disorder with almost complete penetrance (95%) due to loss of functional mutations in two genes: exostosin-1 (*EXT-1*) and exostosin-2 (*EXT-2*) genotypes [1]. The two genes, *EXT1* located at 8q24 and exostosin-2 (*EXT2*) located at 11p11-p12, respectively, have been well characterized [2]. MHE has an estimated prevalence of 1 per 50,000 individuals in Caucasians who are the most common race affected. The incidence in the Western population is only 1.5%, with a male : female (M : F) ratio of 1.5 : 1 [3]. These genes are linked to heparan sulfate (HS) synthesis, but the specific molecular mechanisms leading to the disruption of the cartilage structure and the consequent formation of exostoses is not yet clearly understood [4]. However, HS is an essential component of the body and is present in the growth plates. HS has numerous functions including regulation of the distribution and availability of the growth and signal proteins and their respective interactions [5]. Osteocartilaginous exostoses (Osteochrondromas) arise from the epiphyses of long bones as exophytic lesions. The exostoses are a result of dysplasia in the peripheral part of the growth plate. The bones that are most commonly affected are the lower femur, upper tibia (i.e., around the knee), and upper humerus but may also be found in other bones like the scapula and ilium where they can be missed clinically [6, 7]. Facial bones are not affected as they grow by intramembranous ossification. An osteochondroma is a benign cartilage-capped bony outgrowth which may present as sessile or pedunculated [7]. It is made up of cortex and marrow cavity that are continuous with the host bone. The pedunculated osteochondromas are always directed away from the growth plate and the joint. The most common site for the osteochondromas is the lateral side of the most active growth plate of a long bone. Osteochondromas grow and increase in size in childhood until closure of the growth plate [8].

The osteochondromas tend to become symptomatic around 3 years of age. Most of the affected children will experience pain and restricted joint motion. Some exostoses can also interfere with normal growth plate development, giving rise to limb deformities and eventual limb shortening. Boys are most commonly affected and clinical attention is usually sought when the exostoses compress a nerve or blood vessel and in rare circumstances when there is malignant transformation. In the majority of cases, they complicate as chondrosarcomas. However, other tumor types (osteosarcoma, malignant fibrous histiocytomas, and fibrosarcomas) have also been seen. However these are rare [9]. Malignant transformation has only been reported in the literature only about thirteen times, making it a very rare occurrence. A recent study has also shown that moderate defects or osteochondroma-like outgrowths are present in the cranial base of HME patients [10]. We describe a case of an 11-year-old periadolescent male with MHE and osteoblastic osteosarcoma.

## 2. Case Presentation

**Figure 1:**
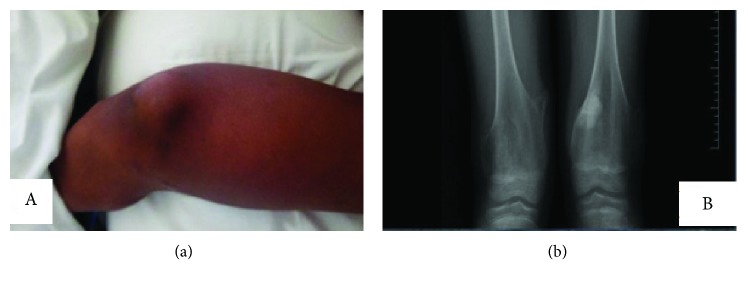
(a) marked swelling and induration of the left distal femur and (b) X-ray shows bone outgrowths (exostoses) on the medial and lateral aspects of the distal femur bilaterally and left metaphyseal widening (AP view).

**Figure 2:**
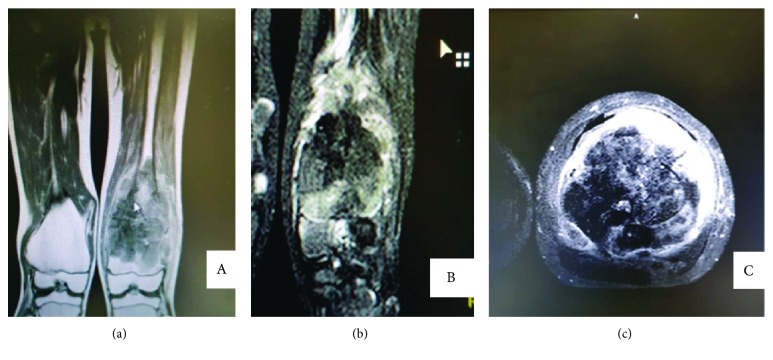
MRI with (a) coronal T2W1 demonstrates heterogeneous signal intensity, (b) coronal T1W1 with contrast, and (c) axial T1W1 with contrast demonstrates moderate heterogeneous enhancement. There were no skip lesions.

**Figure 3:**
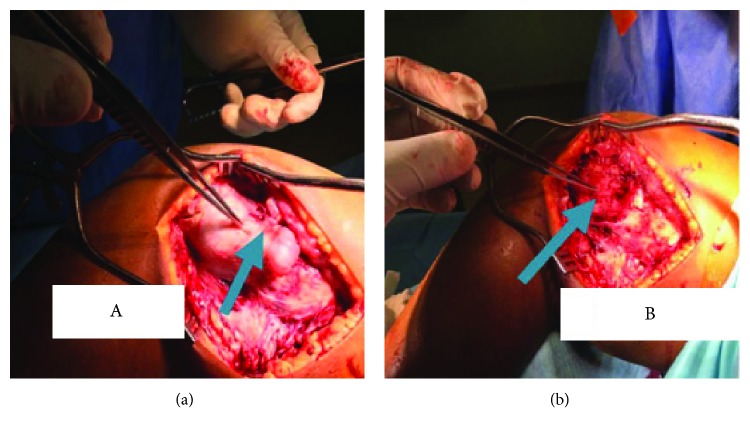
Photograph showing the (a) exostosis and (b) after excision of the exostosis of the left distal femur (see thick blue arrows).

An 11-year-old male Rwandan, a known patient of multiple hereditary exostoses (MHE), presented to our hospital with a 6-month history of unrelenting bone pain despite treatment with NSAIDs; he later developed (two weeks prior to admission) a dull persistent aching pain and swelling of his left lower thigh that was worse at night and unresponsive to morphine. His parents noticed that he had also lost weight and appetite. There was no history of trauma or a fall. He was diagnosed with MHE at age 3 and had been living a relatively normal life and attending school. None of his siblings or any other member of his family had MHE. His past surgical history was unremarkable. On general examination, he was well nourished, had mild pallor of the mucus membranes, no jaundice, lymphadenopathy, or skin rash. All the other parameters were normal. Examination of the musculoskeletal system revealed normal stature except for the curving deformity of the left leg. There were multiple palpable bony swellings bilaterally on the upper humeri and lower femurs. The lesion on the left distal femur was markedly enlarged and tender, with induration, reddening, and limited range of motion of his knee joint (Figure 1(a)). Repeat X-rays confirmed the presence of bony outgrowths (exostoses) on the medial and lateral aspects of the distal femurs bilaterally and left metaphyseal widening common in this condition as had been previously identified when he was diagnosed at age 3, and further investigations of the left distal femur swelling (Figure 1(b)) with magnetic resonance imaging (MRI) revealed a distinct enhancing lesion in the distal aspect of the left thigh at the site of intense swelling and pain. MRI showed a lesion with hypointense signals on T1WI sequences and has heterogeneous signal intensities with moderate and heterogeneous enhancement on T1WI postcontrast study and on T2WI sequences; the lesion had heterogeneous signal intensities (Figures 2(a)–2(c)). At surgery, there was a necrotic lesion, and excision biopsy at the site of the left distal femur exostosis was taken (Figures 3(a) and 3(b)). Hematological evaluation was normal except for a mild leukocytosis. The results of the serum biochemical tests were also normal. We did not do genotyping for EXT-1 and EXT-2 due to lack of facilities.

**Figure 4:**
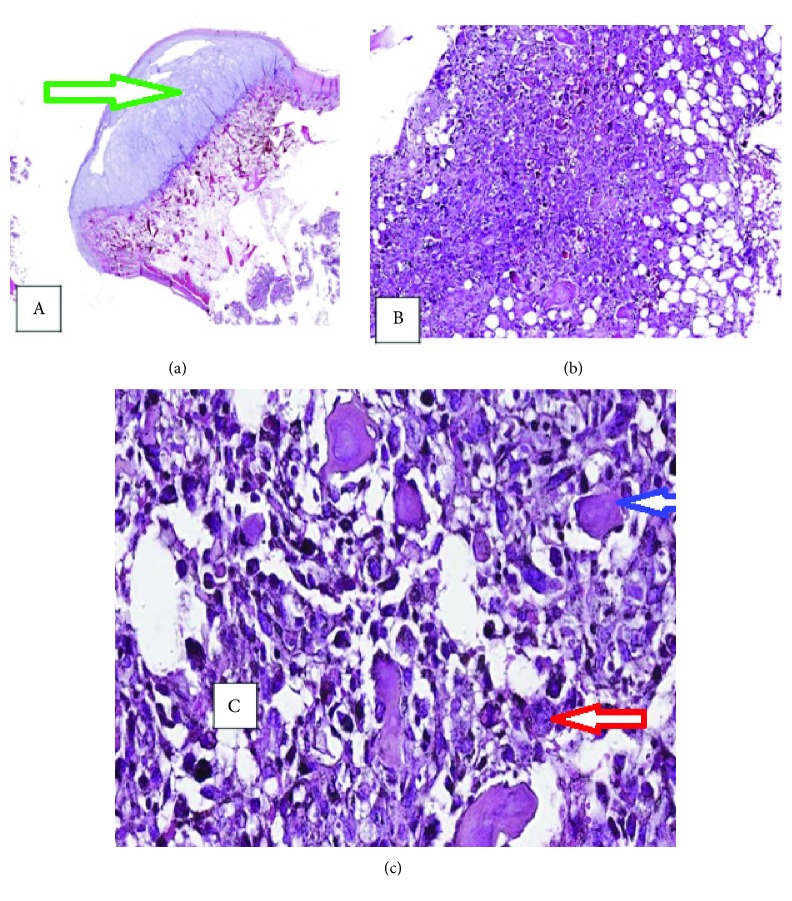
Photomicrographs showing (a) fibrocartilaginous cap (osteochondroma or exostosis-green arrow) (H&E ×20), (b) the tumor-forming osteoid in the marrow and numerous pleomorphic bizarre osteoblasts (H&E ×100), and (c) higher power showing spicules of new bone (osteoid) (blue arrow) among the osteoblasts (red arrow) (H&E ×400).

Histopathological examination revealed a characteristic fibrous cartilaginous cap with a broad base (1.293 mm thick), covering a layer of normal appearing marrow and bone below which was a tumor-forming osteoid, an osteoblastic tumor as evidenced by presence of numerous bone spicules of varying maturity. There was also marked cellular atypia, grossly pleomorphic osteoblasts in the marrow with frequent mitoses. These features were those of a high-grade osteoblastic osteosarcoma. In conclusion, histopathological revealed an osteochondroma with an underlying high-grade osteoblastic osteosarcoma involving the marrow cavity (Figures 4(a)–4(c)).

**Figure 5:**
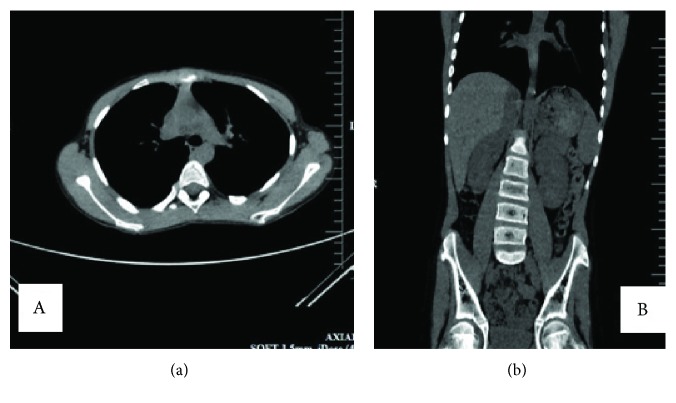
CT scan showing (a) axial section of the chest and pelvis cavity, no metastases detected, and (b) abdomen and chest are normal.

For staging purposes, CT scans of the chest, abdomen, and pelvis were done to investigate any presence of metastatic lesions (Figures 5(a) and 5(b)). There were no metastatic deposits in the lungs, abdomen, and pelvis, and this was confirmed with PET scan in India where the patient was referred for specialized bone tumor treatment including limb salvage therapy. He was reevaluated, and the diagnosis of MHE and osteoblastic osteosarcoma confirmed. Whole body PET scan showed metabolically active disease in the distal left femur 8.6 × 8.1 × 16 cms in dimension with features consistent with osteosarcoma, multiple hereditary exostoses with evidence of skip lesions, loco-regional lymph node involvement, and no distant metastases. Histopathological review confirmed the earlier diagnosis of osteochondroma with osteoblastic osteosarcoma. The conclusion was that the patient had clinically localized disease. The following treatment plan was proposed and instituted: initiation with neoadjuvant chemotherapy followed by limp salvaging surgery and finally adjuvant chemotherapy. He received doxorubicin 35.5 mgs/m2 per day (day 1 and day 2), cisplatin 60 mgs/m2 for 7 days (days 1 and 2), methotrexate 12 gms/m2 per day, etoposide 100 mgs/m2/day for 5 days, and ifosfamide 2.8 gms/m2/day for 5 days followed by Ifosfamide 3 gms/m2/day for 3 days and subsequently pegylated Interferon-*α*2b 0.5 mcg/kg - 1mcg/kg. He completed a total of six cycles of chemoimmunotherapy.

Our patient is still alive and back at school with no evidence of disease after 11 months of treatment and follow-up and continues to be followed up by the oncologist.

## 3. Discussion and Review of Literature

Multiple hereditary exostoses (MHE) with malignant transformation to osteosarcoma has only been reported in thirteen cases in the literature (see Table 1 below detailing the 13 cases) [11–14]. Malignant transformation of osteochondromas rarely occurs in children, and so far, less than five cases have been reported. Three of the five patients had malignant transformation to osteosarcoma: a 12-year-old girl who developed an undifferentiated osteosarcoma with pulmonary metastases at diagnosis, her disease was resistant to chemotherapy so she quickly succumbed and died [11]. The majority of patients with MHE develop malignant complications as adults and the majority will have secondary peripheral chondrosarcoma [15] while the minority develop either osteosarcoma, malignant fibrous histiocytoma, or fibrosarcoma. Recently, an Ewing's sarcoma has also been reported [16]. The diagnosis in our patient was of a high-grade osteoblastic osteosarcoma in the region of the left distal femur at the same site as one of his multiple osteochondromas at only age 11. This is similar to two other cases of a 13-year-old male with a low-grade osteosarcoma and a young girl of 12 years with a high-grade osteosarcoma. Unlike the 12-year-old female, our patient had no evidence of metastases at the time of diagnosis and was referred to a bone tumor reference centre in India for definitive limb salvaging management, further investigations with PET scans in India confirmed no metastases and so he was managed as having clinically localized disease. He is currently on chemotherapy and doing extremely well. The 13-year-old male with a low-grade osteosarcoma fared well with six-cycle treatment of doxorubicin and cisplatin. Like our patient, he presented with similar symptoms of pain and swelling of the leg around the knee joint with joint limitation [13]. Most of the patients begin to present with symptoms (mainly pain and joint restriction) around the time of adolescence when there is rapid bone growth and development. It has also been noted that transformation to osteosarcoma is very rare and has been reported less than 15 times. Normally, malignant transformation takes place at the cartilage cap [9], but in our case, it occurred beneath the stalk of the osteochondroma. This is similar to another patient who presented with an osteosarcoma in the stalk and not the cartilage cap, as would have been expected normally [13].

We can therefore not certainly state that the osteosarcoma arose from the osteochondroma in our patient but it seems more likely that it arose as an independent tumor in the stalk of the osteochondroma not the cap where malignant transformation usually occurs.

Studies have also shown that osteosarcomas tend to develop in the adolescent years [10, 17] when there is rapid bone growth as was the case in our patient, making it also likely that the tumor arose independently. The other risk factor for osteosarcoma that our patient had was chronic bone disease with MHE [18].

It is also thought that malignant transformation of osteochondromas mainly in the cartilage cap develops through a stepwise fashion first into low-grade chondrosarcoma then dedifferentiates into high-grade sarcoma that may eventually become fibrosarcomas or osteosarcomas [19].

It has also been shown that some of the osteosarcomas which are secondary to osteochondromas pathologically show the phases of the stepwise theory by having all kinds of neoplastic tissue such as normal osteochondroma, low-grade chondrosarcoma, and high-grade osteosarcoma [20]. This was not the case in our patient who had low-grade osteochondroma in the cap, below which was an area of normal bone marrow and below the normal marrow area was a high-grade osteoblastic osteosarcoma which was localized to the stalk with no evidence of metastases both at CT scan and PET scan.

Our patient's tumor therefore belongs to the category of the osteosarcomas that arise in the spongeous bone of the stalk, has no relationship with the cartilaginous cap, shows no thickening of the cap, and no neoplastic cartilaginous components. This is similar to that of a 23-year-old male where malignant transformation occurred in the stalk and not the cartilage cap [13].

Regarding the molecular genetics of our patient, there was no family history of MHE, and we were unable to carry out genetic studies to ascertain the presence of mutations in the EXT-1 and EXT-2 genes that have been shown to be the key drivers for the development of MHE [2]. The preliminary diagnosis of MHE is made on radiological diagnosis using plain radiography; the bony outgrowths that typically present on the long bones around the joints of long bones were seen in our patient as has been reported previously [21]. In the background of pain and swelling in our patient, the finding of a distinct enhancing lesion with irregular margins on tissue-contrasted MRI was suggestive of possible malignancy and was able to distinguish the osteochondroma from other bone lesions. This is similar to what other authors have described [22]. Differentiating a benign from malignant tumor on MRI may be challenging, the thickness of the cartilaginous cap can assist with the diagnosis of a chondrosarcoma, when more than 3 cms in a child, it is indicative of a probable chondrosarcoma. This was in contrast to our patient whose cartilaginous cap was only 1.293 mm (normal range 1-2 mm) and the tumor, a high-grade osteoblastic osteosarcoma which arose from the stalk not the cap [23].

The prognosis of patients with MHE that develop osteoblastic osteosarcoma is good when the disease is localized and is managed by a multidisciplinary team of orthopaedic surgeon, radiologist, pathologist, geneticist, and an oncologist. Bone conserving treatment when instituted spares the affected limb as was the case in our patient. Our patient is currently receiving chemotherapy for six cycles with cisplatin and doxorubicin with a great response.

Further management will include insertion of a bone prosthesis to ensure bone strengthening and stability at the tumor site after chemotherapy.

## 4. Conclusion

We present an unusual case of a secondary osteoblastic osteosarcoma that developed beneath the stalk of an osteochondroma in a patient with multiple hereditary exostoses.

## Figures and Tables

**Table 1:** The table shows 13 cases with MHE and malignant transformation.

Cases	Age (yrs), sex	Clinical summary	Imaging findings	Malignancy	Reference
1	11, M	Family h/o MHE, 9 mos mild pain and worsening limp with 2 mos palpable enlarging pelvic enchondromas	10 × 12 × 12 cms Sessile osteochondroma Left posterior pelvic ilium	Low-grade chondrosarcoma	Schmale GA et al. Sarcoma 2010, Article ID 41705, 7 pages, 2010
2	13, M	Family h/o MHE, 6 mos h/o of painless enlarging osteochondroma of left distal femur	10 × 10 × 7 cms lesion with sclerotic margin at distal femoral diaphysis, displaying obvious growth	Low-grade chondrosarcoma	Schmale GA et al. Sarcoma 2010, Article ID 41705, 7 pages, 2010
3	35, M	Pelvic pain, difficulty in urination, and defecation	Large mass with pelvic outlet demonstrating 10 × 9.4 × 14 cms	Chondrosarcoma	http://Radiopaedia.org
4	18, F	Bilateral bone knee deformities and associated pain	Multiple sessile and pedunculated exostoses bilaterally, peripheral permeation and heterogeneous appearance, MRI: large osteochondroma with irregular cartilage cap 10 mm	Low-grade chondrosarcoma	Vlok SCS et al. SA J Radiol 2014; 18(2):1-5
5	60, F	Known Ollier's disease with increased swelling and pain in the right middle finger	Multiple enchondromas bilaterally right hand of metacarpals and phalanges with expansile growth in the right middle finger	Grade II chondrosarcoma	Vlok SCS et al. SA J Radiol 2014; 18(2):1-5
6	14, F	Tumor on L coxa	Ostedestructive lesion in the superior 3rd of the left femur	Grade I chondorsarcoma	Gomes ACN et al. Radiol Bras 2006; 39(6): 449-451
7	30, F	Multiple exostoses over the right elbow, the left knee, and cervical body of C6 on the right. Positive family h/o 3 mos sudden rounded hard lesion, Right cervical region	MRI: rounded lesion arising from right articular mass of C6, hyperintense on T1 weighted images with a small enhancing nodule	Grade I chondrosarcoma	Landi A et al. J Solid Tumors 2012; 2(3): 63-70
8	14, F	Increasing pain and swelling of the left knee joint, 2 yrs	Juxtacortical tumor, irregularly and heavily ossified with focal lucent areas at proximal end of the left tibia. CT: large mass with areas of irregular mineralization	Chondroblastic osteosarcoma	Nojima T et al. 1991; 62(3):290-292
9	19, F	Family h/o MHE		Spindle cell sarcoma	Matsuno et al. 1988. J Bone Joint Surg. Am. 70 : 137-141
10	29, M	Family h/o MHE		Spindle cell sarcoma	Matsuno et al. 1988. J Bone Joint Surg. Am. 70 : 137-141
11	48, M	Family h/o MHE, abdominal pain and mass, erectile dysfunction, urinary symptoms	Mass seen in pelvis on CT, MRI 16 × 12 × 13 cms arising from the left pubic bone, which deformed the bladder and sigmoid colon	Undifferentiated chondrosarcoma	Willms et al. 1997. Int. Orthopead 21: 133-136
12	23, M	Pain and swelling on the left proximal tibia, later joint limitation and pain, swelling in posteromedial aspect of the proximal tibia	Large-based sessile osteochondroma which involved almost the whole bone	Osteosarcoma	Engel EE et al. 2012. Genet. Mol. Res. 11(1): 448-454.
13	60, F	Bilateral femoral shaft fractures after falling out of wheel chair		Chondrosarcoma	Rupp M et al. 2016. Orthop Rev 8(3):1-4

mos: months; h/o: history of; MHE: multiple hereditary exostoses; cms: centimeters; MRI: magnetic resonance imaging; CT: computerized tomography.
